# How Bones Heal

**Published:** 1875-03

**Authors:** 


### HOW BONES HEAL.

Directly after the occurrence of the fracture, there is an effusion of blood around the broken ends of the bone. The blood soon gives place to a plasma, which becomes organized, and changes first into a cartilage, and then into bone. This newly formed bone takes the shape of a ferule, surrounding the fractured ends and holding them together. It is of elliptic form, its greatest diameter and strongest point being around the ends that are to be kept in place. It is completed in six or eight weeks, according to the age and vigor of the patient. Then it is said—in common language—that the bone has "knit together." But this is not strictly true; the fractured ends are not yet united—they are simply held in place by the ring of new bone, which is nature's splint, and which now supersedes the splints and bandages of the surgeon. This ferule is called "the provisional callus," a term significant of its nature and its temporary office. For, as soon as the reparative powers of the system have had time to re-establish the original union of the parts, nature's splint can be dispensed with, and is accordingly absorbed, and passes away. This work is accomplished in from six to twelve months. But this ferule, or ring, is not always formed at the seat of a fracture; it never occurs in fractures of the skull, for the bony projection from the inner surface would produce pressure on the brain, and interfere with the cerebral functions. Other bones are thus held in place while the union is being accomplished; but nature spares the brain from the pressure of callosities by securing the union of the bones of the skull—which are not subject to strain during the healing process—by a direct cementing process.

"Utility."—Our attention has lately been attracted to an adjustable table, designed especially to be used by ladies as a work-table. It can be raised or lowered in an instant, and can be folded up and packed into very small space. It not only takes the place of the lap-boards in common use among ladies, but answers all purposes for which small-sized tables are employed. We are so well pleased with this adjustable table that we propose to describe it fully next month, and illustrate our article with attractive cuts. These tables are made of different styles, by Messrs. Lambie & Sargent, 793 Broadway, New York, who will mail descriptive circulars to all who desire them.

---

Enameled Kettles.—The silicious mixture fused upon iron to form an enamel for kettles and saucepans, frequently contains lead, which is used as a flux. Boiling vinegar and many other vegetable acids are sufficient to dissolve the coating and extract a dangerous portion of the lead. It is safer to use plain cast-iron, in spite of its tendency to oxydize and discolor the materials cooked.

---

Pneumonia and other inflammatory diseases, continue to be the most frequent causes of death in New York city. During the eleven weeks since the very cold weather began, there have been 867 deaths charged to pneumonia, and 339 to bronchitis, in a total mortality of 6,712.

---

How we made Bread.—The second installment of the interesting story, "Our Summer at Maplewood," appears in this number, and will be especially attractive to our lady readers.

